# Dysregulation in Subcellular Localization of Myelin Basic Protein mRNA Does Not Result in Altered Myelination in Amyotrophic Lateral Sclerosis

**DOI:** 10.3389/fnins.2021.705306

**Published:** 2021-09-01

**Authors:** Samantha K. Barton, Jenna M. Gregory, Bhuvaneish T. Selvaraj, Karina McDade, Christopher M. Henstridge, Tara L. Spires-Jones, Owen G. James, Arpan R. Mehta, David Story, Karen Burr, Dario Magnani, Adrian M. Isaacs, Colin Smith, Siddharthan Chandran

**Affiliations:** ^1^Florey Institute of Neuroscience and Mental Health, The University of Melbourne, Melbourne, VIC, Australia; ^2^Euan MacDonald Centre for MND Research, The University of Edinburgh, Edinburgh, United Kingdom; ^3^Centre for Clinical Brain Sciences, The University of Edinburgh, Edinburgh, United Kingdom; ^4^UK Dementia Research Institute at The University of Edinburgh, The University of Edinburgh, Edinburgh, United Kingdom; ^5^Centre for Discovery Brain Sciences, The University of Edinburgh, Edinburgh, United Kingdom; ^6^UK Dementia Research Institute at UCL, Faculty of Brain Sciences, University College London, London, United Kingdom; ^7^Department of Neurodegenerative Disease, UCL Queen Square Institute of Neurology, University College London, London, United Kingdom; ^8^Department of Neurodegenerative Disease, UCL Institute of Neurology, London, United Kingdom

**Keywords:** oligodendrocytes, RNA trafficking, myelination, ALS, myelin basic protein

## Abstract

Pathological hallmarks of amyotrophic lateral sclerosis (ALS), including protein misfolding, are well established in oligodendrocytes. More recently, an RNA trafficking deficit of key myelin proteins has been suggested in oligodendrocytes in ALS but the extent to which this affects myelination and the relative contribution of this to disease pathogenesis is unclear. ALS autopsy research findings showing demyelination contrasts with the routine clinical-pathological workup of ALS cases where it is rare to see white matter abnormalities other than simple Wallerian degeneration secondary to widespread neuronal loss. To begin to address this apparent variance, we undertook a comprehensive evaluation of myelination at an RNA, protein and structural level using human pathological material from sporadic ALS patients, genetic ALS patients (harboring *C9orf72* mutation) and age- and sex-matched non-neurological controls. We performed (i) quantitative spatial profiling of the mRNA transcript encoding myelin basic protein (*MBP*), (ii) quantification of MBP protein and (iii) the first quantitative structural assessment of myelination in ALS post-mortem specimens by electron microscopy. We show no differences in MBP protein levels or ultrastructural myelination, despite a significant dysregulation in the subcellular trafficking of *MBP* mRNA in ALS patients compared to controls. We therefore confirm that whilst there are cell autonomous mRNA trafficking deficits affecting oligodendrocytes in ALS, this has no effect on myelin structure.

## Introduction

Oligodendrocyte pathology has been demonstrated in ALS post-mortem tissue (Kang et al., 2013; Philips et al., 2013; Lorente Pons et al., 2020); however, the relevance and/or contribution of this pathology to disease pathogenesis is relatively understudied. Oligodendrocytes provide support to neurons through two key roles: myelination and metabolic support (Saab et al., 2013). With regards to myelination, previous studies have shown demyelination in ALS autopsy samples (Kang et al., 2013; Philips et al., 2013). These findings are in contrast to what is seen commonly in routine neuropathology practice, where it is rare to observe demyelinating lesions in ALS patient material other than Wallerian degeneration secondary to neuronal loss (Kang et al., 2013). Furthermore, MRI imaging findings are most consistent with white matter abnormalities that reflect neuronal loss and axonal degeneration (Grolez et al., 2016; Martin et al., 2016; Pallebage-Gamarallage et al., 2018; Querin et al., 2019), rather than demyelination *per se*.

Myelin basic protein (MBP) is one of several proteins that is translated locally in the terminal processes of oligodendrocytes and is a major constituent in myelin. Local translation is especially important in cells with multiple, often long processes, making these cells reliant on adequate mRNA trafficking from the cell body to the translation machinery at the terminal processes. Errors in neuronal axonal transport, and more specifically mRNA trafficking and stability, have been implicated in ALS through cell, and potentially non-cell, autonomous mediated mechanisms (Lin et al., 1998; Chang et al., 2008; Bilsland et al., 2010; Swarup et al., 2011; Millecamps and Julien, 2013; Fallini et al., 2016). Of relevance to impaired RNA homeostasis, over 95% of ALS patients display pathological accumulation of TDP-43, an RNA-binding protein that binds to 30% of the human transcriptome (Neumann et al., 2006; Freibaum et al., 2010; Ling et al., 2013). Indeed, dysregulated RNA metabolism is also observed in ALS due to *C9orf72* hexanucleotide repeat expansion (Prudencio et al., 2015), the most common known genetic cause of ALS. Disrupted RNA trafficking and metabolism has been demonstrated in neurons in ALS and the relevance of these findings to oligodendrocytes, which not only display the hallmark pathologies of ALS as well as rely on local translation of many RNAs involved in myelination and metabolism, is becoming clearer through the examination of post-mortem patient samples (Khalil et al., 2018; Lorente Pons et al., 2020). Indeed, many known binding partners of TDP-43 have been implicated in oligodendrocyte function. These include *MBP* mRNA that is trafficked by heterogeneous nuclear ribonucleoproteins (hnRNP) A2/B1 and A3 (Müller et al., 2013); hnRNP A2/B1 and hnRNP A3 are also sequestered by RNA foci transcribed from the GGGGCC repeat (Kapeli et al., 2017). It has been hypothesized recently that oligodendrocyte pathology may be contributing to ALS pathogenesis through deficits in mRNA trafficking and that, in some cases, these effects may exceed neuronal pathology (Lorente Pons et al., 2020). We have also shown previously that up to a fifth of ALS cases (sporadic and genetic) examined at post-mortem have a purely glial pathological signature, with little if any neuronal pathology in non-motor brain regions (Gregory et al., 2019). These findings raise the possibility of a role for cell autonomous glial pathology such as myelination deficit due to disrupted MBP mRNA trafficking in both sporadic and genetic ALS.

Against this background, we have undertaken a comprehensive study of human pathological material with a focus on MBP and myelination from sporadic ALS patients, *C9orf72* patients and age- and sex-matched non-neurological controls. Specifically, we performed (i) quantitative spatial profiling of the *MBP* mRNA transcript to understand subcellular localization using a modified *in situ* hybridization technique called BaseScope^TM^, which allows for the identification of single mRNA transcripts at single cell resolution, (ii) a comprehensive quantification of MBP protein and (iii) a quantitative structural assessment of myelination assessed through both immunohistochemistry and electron microscopy.

## Materials and Methods

### Human Post-mortem Tissue—Acquisition

Human brain tissue was provided by the Medical Research Council (MRC) Edinburgh Brain and Tissue Bank. Patients were identified as having a diagnosis of ALS with further classification as to whether the disease was sporadic or familial (with genetic screening identifying the pathological mutation); patient details are listed in Supplementary Table 1. Age-matched controls were chosen from the Lothian Birth Cohort 1936 (LBC1936), a cohort who have detailed cognitive assessment throughout life, and were listed as negative for ALS diagnosis and TDP-43 pathology. We used LBC1936 control subjects (*n* = 3–5; control) ALS patients with sporadic disease (*n* = 3–5; sALS) and ALS patients harboring a *C9orf72* HRE (*n* = 3–5, C9 ALS). All post-mortem tissue was collected via the Edinburgh Brain Bank (ethics approval from East of Scotland Research Ethics Service, 16/ES/0084) in line with the Human Tissue (Scotland) Act. Use of human tissue for post-mortem studies has been reviewed and approved by the Edinburgh Brain Bank ethics committee and the Academic and Clinical Central Office for Research and Development (ACCORD) medical research ethics committee (AMREC).

At autopsy, brains were sectioned coronally and then regions of interest from the right and left hemispheres were fixed in 10% formalin, with complementary regions from the left hemisphere snap frozen in liquid nitrogen. Separately, small blocks were excised and fixed for electron microscopy. Formalin fixed blocks underwent dehydration in ethanol titrations (70–100%), were put through three xylene washes before being infiltrated with paraffin wax. Blocks were then embedded in paraffin wax and sectioned at 4 μm onto Superfrost slides. The region of interest used for this study was the standardized Brodmann area (BA)4 allowing analysis of the primary motor cortex white matter. For g-ratio analysis, samples of anterior corpus callosum and subcortical white matter (BA6/8) were processed for EM as previously described (Kay et al., 2013). Briefly, tissue was dissected into small blocks and fixed in 4% paraformaldehyde and 1% glutaraldehyde in 0.1 M PB for 48 h. Once fixed, the blocks were washed twice in 0.1 M PB and then incubated in 1% osmium tetroxide for 30 min and then negatively stained with 1% uranyl acetate before dehydration through titrations of ethanol and propylene oxide; blocks were then embedded in Durcupan resin for 48 h at 56°C. Ultrathin sections (70 nm) were cut from selected areas, stained in Uranyl Acetate and Lead Citrate then viewed in a JEOL JEM-1400 Plus TEM. Two blocks were analyzed per patient (*n* = 3 patients per group) per region and images were taken using a GATAN OneView camera. The white matter was identified as regions with densely myelinated axons and all of our blocks were entirely white matter enabling us to utilize the entire grid; we commenced imaging in the top left corner of the grid and imaged clockwise until at least 100 axons per patient were imaged. We aimed for 150 countable analyzable axons per block and this varied between 10 and 20 grids being imaged, depending on the integrity of the tissue. G-ratios were calculated using Fiji (ImageJ) by measuring the ratio of inner axonal diameter to outer axonal diameter.

### Human Post-mortem Tissue—BaseScope^TM^

BaseScope^TM^ probes were designed and constructed by ACD for three transcripts of interest: the oligodendrocyte specific mRNA transcripts human myelin basic protein (*MBP*), human carbonic anhydrase II (*CAII*), as well as C9 RNA foci (BA-GGGGCCn-3zz-st; ACD #704181). Both the *MBP* and *CAII* probes were designed to amplify all transcript variants for both genes to allow extensive coverage of expression.

For all probes, BaseScope^TM^ RED Reagent Kits were used and assays were run according to guidelines provided. Briefly, sections were dewaxed and rehydrated, blocked for endogenous peroxidases and then antigen retrieval was carried out using the ACD pre-treatment reagent. Protease III was used (30 min; 40°C) before incubation with the probe (2 h; 40°C). Slides probed with the C9 RNA foci probe (Supplementary Data) were treated with 800 U/ml DNase (30 min; RT) after Protease III but prior to probe incubation, as published previously (Mehta et al., 2020). Each Amp reagent was used as per kit instructions; incubations at 40°C were conducted using the HybEZ II Oven (ACD). Following final amplification with Fast Red (Fast Red incubation time was 10 min for MBP and CAII probes and 2 min for C9 probe), slides were counterstained in hematoxylin and then left to dry prior to being cleared in xylene and cover-slipped.

Two sections of motor cortex were used per patient (one from each hemisphere) with analyses limited to the white matter. Slides were scanned using a Nanozoomer (Hamamatsu) and ten fields of view were taken per section at 20x magnification using NDP.view 2 imaging software (Hamamatsu) and analyzed in Fiji (ImageJ). The MBP mRNA was quantified in three ways: (1) the density of nuclei with 7 or more transcripts were counted (nuclear expression should be low because MBP is trafficked for local translation); (2) the density of nuclear aggregations of MBP transcripts whereby more than half the nucleus is occupied by transcripts; and (3) density of cytoplasmic aggregations of MBP transcripts whereby 5 or more transcripts were clustered together in the perinuclear space. CAII mRNA counts were ranked based on a scoring system whereby 0 = no aggregates, 1 = one aggregate, 2 = between one and five aggregates and 3 = more than five aggregates in the whole white matter area per section (Supplementary Data).

### Human Post-mortem Tissue—Neuropathological Analysis

For histological staining, sections were de-waxed and rehydrated prior to staining in Luxol Fast Blue overnight at 40°C. The stain was differentiated in 0.1% lithium carbonate prior to dehydration, clearing and coverslipping. For immunostaining (Supplementary Data), the Novolink Polymer detection system was used to stain for phospho-TDP-43 (s409-410) (Cosmo #TIP-PTD-M01; 1:4,000) and p62 (BD Biosciences #610833; 1:2,000). Following DAB chromagen, the sections were counterstained with hematoxylin. Slides were scanned using a Nanozoomer (Hamamatsu) and representative images extracted using NDP.view 2 (Hamamatsu). Assessors were blinded to all demographic and clinical information. For immunostaining of the dipeptide repeat proteins (DPRs) GA (Everest Biotech G2 CUST05049; 1:200) and GP (Everest Biotech G2 CUST05048; 1:100), sections were manually stained without the Novolink system but still with DAB chromagen and hematoxylin counterstain. We were only able to optimize antibodies for these two DPRs in our tissue. Glial identification was based on a well-established morphological criteria and carried out by a trained neuropathologist.

### Human Post-mortem Tissue—Biochemical Analysis

From frozen blocks of motor cortex, white matter was dissected and used for RNA and protein extraction. The samples were homogenized in their respective buffers for RNA or protein isolation.

For RNA extraction, the RNeasy lipid tissue mini kit (Qiagen) was used according to the guidelines provided. RNA concentration was measured using a spectrophotometer (DeNovex) and cDNA synthesized for quantitative real time PCR (qPCR). The gene of interest was *MBP* (F: 5′-CTTCTTTGGCGGTGACAGG-3′, R: 5′-CGGGGTTTTCATCTTGGGTC-3′) and housekeepers *18S* (F: 5′-GTAACCCGTTGAACCCCATT-3′, R: 5′-CCATCCAAT CGGTAGTAGCG-3′) and *β-actin* (F: 5′-GTTACAGGAAG TCCCTTGCCATCC-3′, R: 5′-CACCTCCCCTGTGTGGACT TGGG-3′) were used for normalization. The MBP primers were designed to span exon 2 and 3 of the transcript variants that encode the 18.5 kDa protein isoform; this is the most abundant protein that is translated locally in oligodendrocytes. Gene expression of *CAII* was also measured using qPCR (F: 5′-TGGTCATGCTTTCAACGTGG-3′, R: 5′-CCATCAAGTGAACCCCAGTG). The method of analysis employed for the qPCR data was the ΔΔCT method.

For protein extraction, tissue was homogenized in RIPA buffer (50 mM Tris HCl pH 7.4, 0.1% SDS, 0.5% sodium dioxycholate, 1% Triton-X, 150 mM NaCl, 2 mM EDTA, protease inhibitor, phosphatase inhibitor). Samples were incubated on ice and then centrifuged at 15,700 × *g* for 5 min at 4°C; the supernatant was transferred to a fresh Eppendorf. Protein concentration was assessed by a BCA protein assay (Pierce), then Laemmli buffer was added (125 mM Tris HCl pH 6.8, 4% SDS, 4% β-mercaptoethanol, 20% glycerol and 0.004% bromophenol blue) and samples were boiled for 10 min at 99°C. For the western blots, protein (20 μg for SOX-10 and 1 μg for MBP) was run on 4–20% SDS/Poly acrylamide gel electrophoresis (Invitrogen) for 2.25 h at 90 V and blotted onto a 0.45 μm polyvinylidene fluoride membrane at 20 V with a transfer time of 1 h 20 min. Membranes were blocked in 5% milk in TBS-T and probed with either MBP (1:1,000; #05-675 Merck) or SOX10 (1:2,500; Ab155279 Abcam) (and β-actin as a housekeeping protein (1:10,000; #A2228 Sigma Aldrich)) overnight at 4°C, appropriate horse radish peroxidase-tagged secondary antibodies (1:5,000; Dako) at room temperature for 2 h and then developed using ECL (GE Healthcare). Blots were scanned and densitometry analysis performed using Fiji (Image-J). Full lane representations are included (see Supplementary Figure 5).

### Statistical Analyses

Data are presented as mean ± SEM. All outputs were analyzed using a one-way ANOVA and Dunnett’s multiple comparisons test for *post hoc* comparisons comparing sALS and C9 ALS groups to control. ^*^ indicates *p* < 0.05.

## Results

### Oligodendrocytes in the Motor Cortex White Matter of Patients With ALS Display MBP RNA Trafficking Deficits

**FIGURE 1:**
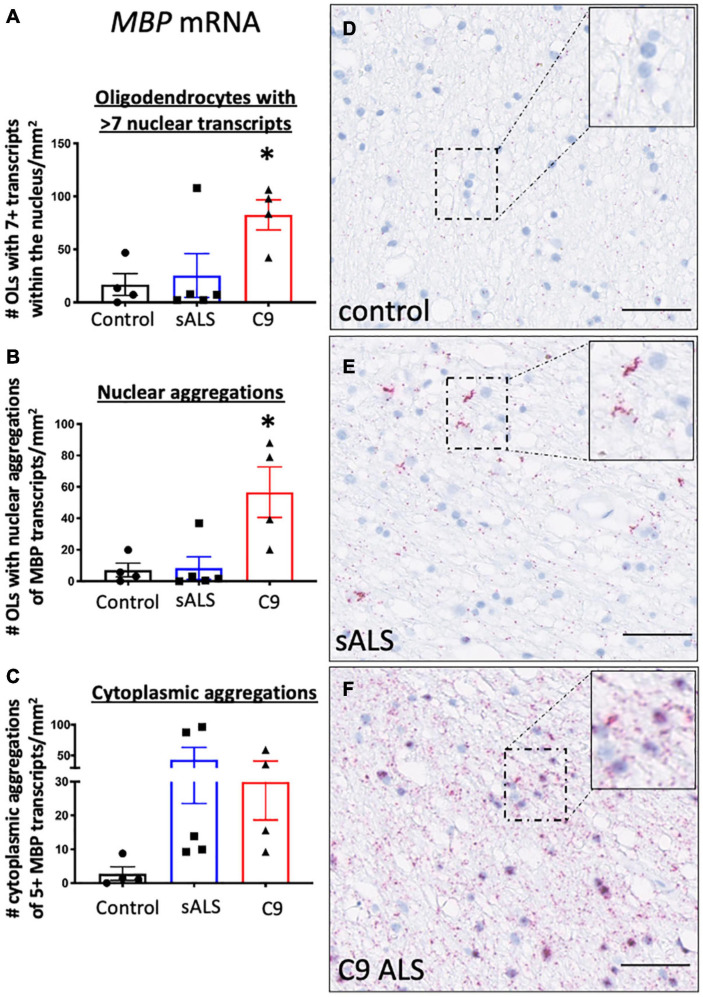
RNA trafficking of myelin basic protein (*MBP)* is dysregulated in oligodendrocytes. MBP mRNA transcript localization is altered in sporadic ALS (sALS) patients and ALS patients with a C9orf72 mutation (C9 ALS) compared to controls. **(A)** Graph demonstrating number of oligodendrocytes (OLs) with 7 or more nuclear *MBP* transcripts. Data show that *C9orf72* ALS cases had a higher proportion of nuclei with > 7 transcripts (*p* < 0.05 compared to control; *n* = 4–5 cases for each genotype). **(B)** Graph demonstrating number of oligodendrocytes (OLs) with aggregations *of MBP* transcripts. Data show that C9orf72 cases have more nuclear aggregations *of MBP* transcripts (*p* < 0.05 compared to control; *n* = 4–5 cases for each genotype). **(C)** Graph demonstrating number of cytoplasmic aggregations of *MBP* transcripts. Some sALS and C9orf72 cases have more cytoplasmic aggregations than control individuals (*n* = 4–5 cases for each genotype). **(D)** Representative image from a control case showing MBP positive transcripts within the cytoplasm and cellular processes. **(E)** Representative image of a sALS case showing prominent cytoplasmic aggregates of transcripts. **(F)** Representative image from a *C9orf72* case demonstrating prominent nuclear and cytoplasmic aggregations of transcripts. * denotes *p* < 0.05. Scale bar = 50 μm.

Given recent findings suggesting protein pathology in oligodendrocytes in ALS is associated with an RNA trafficking deficit (Lorente Pons et al., 2020), we first evaluated hallmark protein pathologies in oligodendrocytes. In support of previous findings (Neumann et al., 2006; Lorente Pons et al., 2020), we identified TDP-43 and p62 pathology in both sporadic and *C9orf72* patients (Supplementary Figure 1) as well as *C9orf72* specific pathologies including RNA foci and dipeptide repeat protein accumulation (GP and GA; Supplementary Figure 2). To assess RNA trafficking, we measured expression of *MBP* mRNA, encoding one of the most abundant myelin proteins and a trafficked mRNA for local translation, using BaseScope^TM^. Qualitative assessment of the distribution of the oligodendrocyte specific mRNA transcript *MBP* in control samples showed evenly dispersed *MBP* mRNA localized to the neuropil consistent with localization within cellular process implying adequate mRNA transport to terminal processes (Figure 1A). In contrast, samples from sALS (Figure 1B) and *C9orf72* patients (Figure 1C), revealed *MBP* mRNA accumulation in mRNA inclusions in both the nucleus and cellular processes. Quantification showed an increased number of oligodendrocytes with seven or more *MBP* mRNA transcripts in *C9orf72* ALS patients compared to controls (*p* = 0.043; Figure 1D) as well as an overall increase in the number of oligodendrocytes with aggregations of *MBP* mRNAs (whereby more than half the nucleus was occupied by *MBP* transcripts; Figure 1E) compared to controls (*p* = 0.013). Finally, perinuclear aggregates of *MBP* transcripts (whereby 5 or more *MBP* transcripts were clustered together) were present in sALS and *C9orf72* ALS cases but the variability amongst cases meant this did not reach statistical significance (*p* = 0.19; Figure 1F).

To determine if the RNA trafficking deficit was specific to *MBP* or affected other oligodendrocyte-specific mRNAs not involved in myelin production as previously reported (Lorente Pons et al., 2020) we next examined the distribution of *CAII*. *CAII* expression is abundantly expressed in oligodendrocytes (Komoly et al., 1987; Lorente Pons et al., 2020) and is a known binding partner of monocarboxylate transporter 1 (MCT1), the oligodendrocyte lactate transporter. A higher proportion of sALS (Supplementary Figure 3B) and *C9orf72* ALS (Supplementary Figure 3C) cases had aggregations of *CAII* mRNA transcripts compared to control cases (Supplementary Figure 3A) but this did not reach statistical significance (*p* = 0.17; Supplementary Figure 3D). There was also no statistical difference in total *CAII* gene expression between groups as measured using qPCR (Supplementary Figure 4).

### MBP mRNA Aggregation Does Not Affect Myelination

**FIGURE 2:**
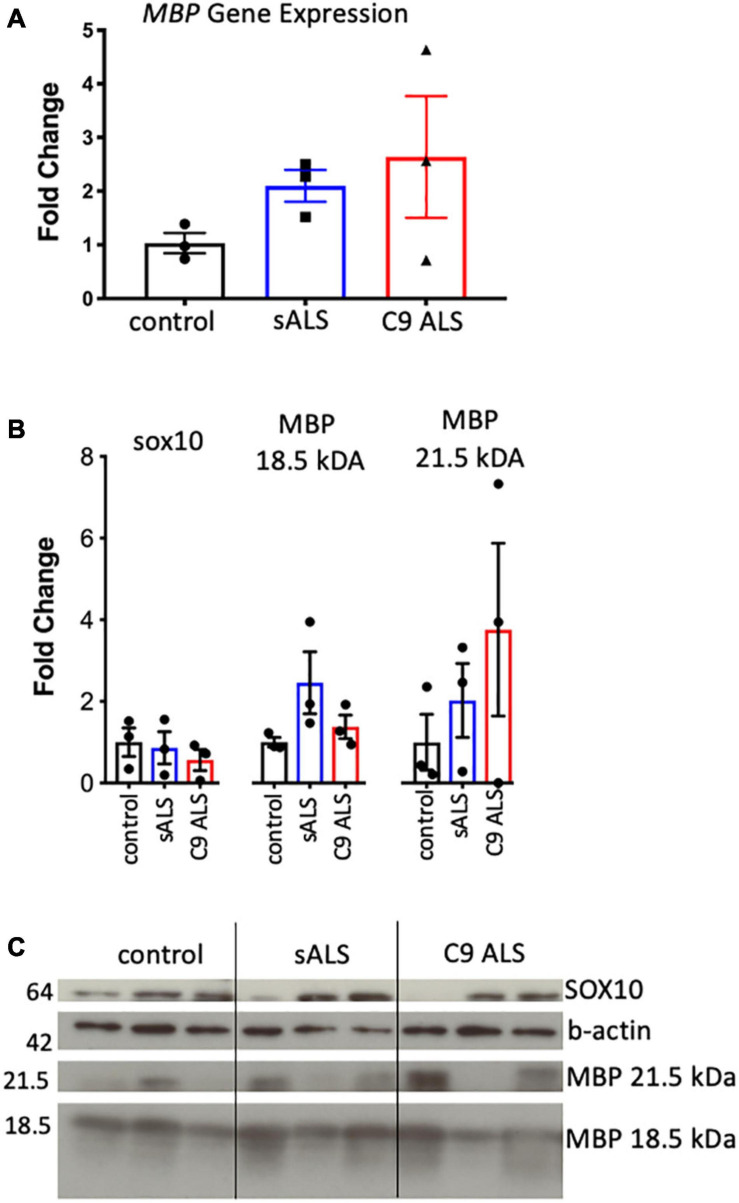
MBP mRNA and protein levels were variable and not relative to RNA trafficking dysfunction. **(A)** Graph showing *MBP* gene expression in whole tissue homogenate of motor cortex white matter showing no statistically significant difference in expression between cases (*n* = 3 for each genotype). **(B)** Graph showing SOX10 and protein expression of two MBP protein isoforms in whole tissue homogenate of motor cortex white matter showing variable but not significantly different protein expression between cases (*n* = 3 for each genotype). **(C)** Western blot that was graphed in b showing SOX10 and MBP protein expression.

Having established disruption of *MBP* mRNA trafficking in *C9orf72* and sALS cases, we next wanted to ascertain whether this had an effect on the total amount of *MBP* mRNA and protein. We used qPCR to measure abundance of *MBP* mRNA, with primers designed to amplify the splice variant commonly trafficked for local translation, and found variable expression in ALS cases but no statistically significant difference (Figure 2A). This variability was also apparent with respect to total MBP protein levels when assessing both the 18.5 and 21.5 kDa isoforms (Figures 2B,C).

**FIGURE 3:**
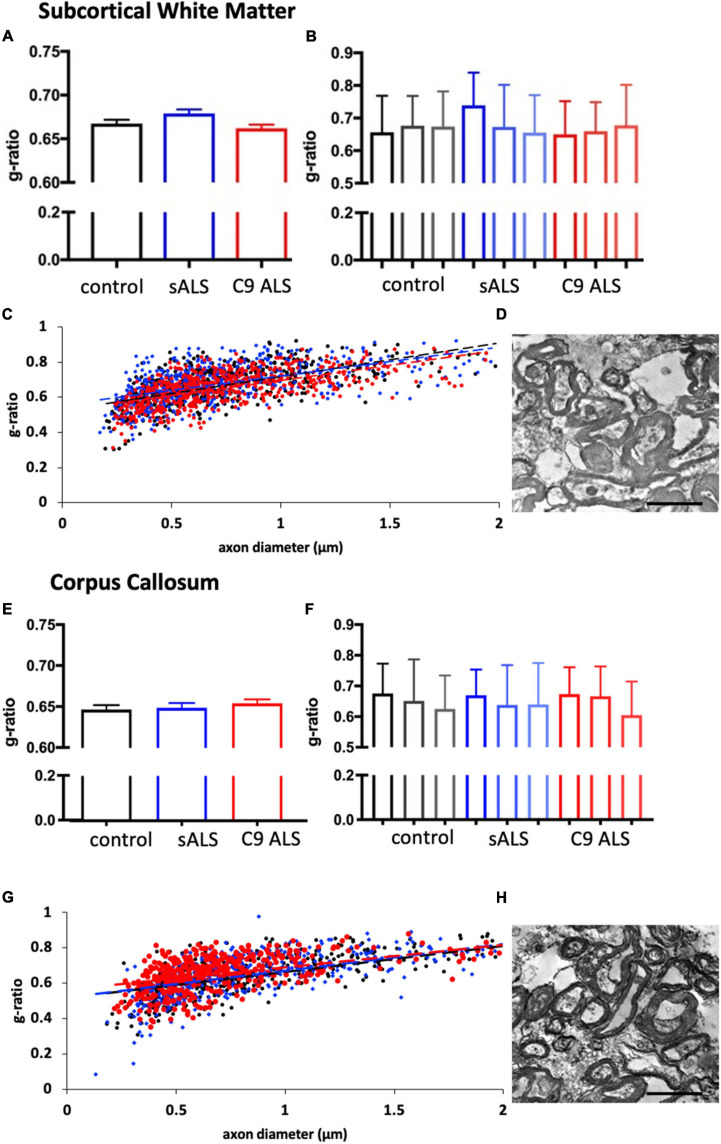
Myelin thickness is not altered in ALS patients. **(A)** Graph showing the g-ratio calculated for sALS and *C9orf72* ALS patients and compared to control patients in the BA6/8 subcortical white matter showing no difference between cases; *n* = 3 for each genotype. **(B)** Graph showing the g-ratios for each individual sALS and *C9orf72* ALS patient compared to controls in the BA6/8 subcortical white matter. **(C)** Graph showing g-ratio respective to axon diameter for control patients (black), sALS patients (blue) and *C9orf72* patients (red) in the BA6/8 subcortical white matter. **(D)** Representative electron micrograph from the motor cortex white matter of a C9 ALS patient. **(E)** Graph showing the g-ratio calculated for sALS and *C9orf72* ALS patients and compared to control patients in the corpus callosum showing no difference between cases; *n* = 3 for each genotype. **(F)** Graph showing the g-ratios for each individual sALS and *C9orf72* ALS patient compared to controls in the corpus callosum. **(G)** Graph showing g-ratio respective to axon diameter for control patients (black), sALS patients (blue) and *C9orf72* patients (red) in the corpus callosum. **(H)** Representative electron micrograph from corpus callosum of a C9 ALS patient Scale bar = 7 μm.

**FIGURE 4:**
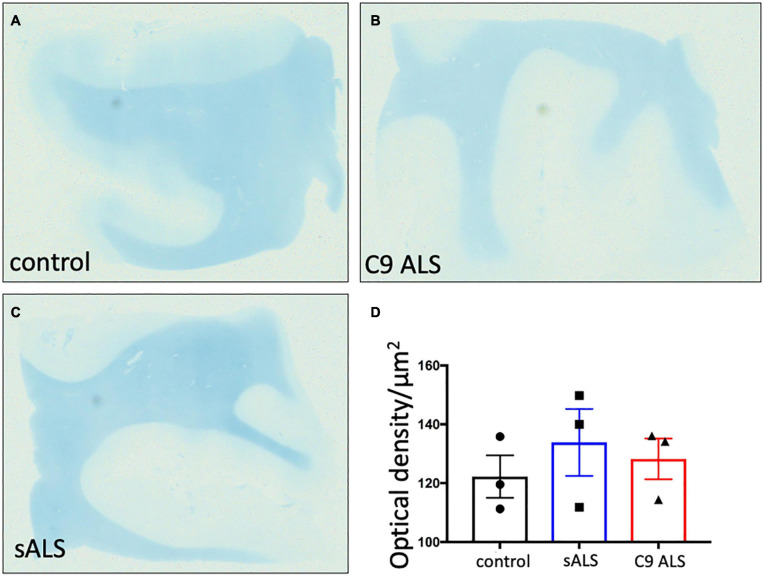
There is no difference in myelin density between groups. **(A–C)** Representative images of Luxol Fast Blue histological staining of the motor cortex (BA4) showing no difference in staining intensity between groups. **(D)** Graph showing no difference in the optical density in sALS and C9 ALS patients compared to controls. Optical density was calculated by measuring the average intensity of staining of the entire white matter region per section; a higher optical density correlates to worse myelination.

To more comprehensively assess myelination, we next undertook luxol fast blue (LFB) tinctorial staining of the motor cortex white matter as well as electron microscopy (EM) of both the motor cortex and corpus callosum of ALS patient human post-mortem tissue compared to age- and sex- matched controls. No difference was found in the ultrastructural evaluation of myelination using gold standard g-ratio measurements in either the subcortical white matter adjacent to the motor cortex (Figures 3A–D) nor the corpus callosum (Figures 3E–H). This was further confirmed using LFB staining (Figures 4A–D). Whilst our findings don’t rule out altered nodal morphology nor the presence of myelin blebbing, our findings show that there is no structural change in the compaction of myelination in the cortex of ALS patients, despite *MBP* disrupted mRNA trafficking.

## Discussion

RNA trafficking is disrupted in motor neurons in ALS and this has been attributed to common ALS pathologies, including TDP-43 aggregation, resulting in microtubule instability, axonal transport defects and impaired synaptic activity (Khalil et al., 2018; Lorente Pons et al., 2020). It is well established that many mRNAs are also locally translated in oligodendrocytes and oligodendrocytes harbor common ALS pathologies, yet there has been limited study of RNA trafficking in oligodendrocytes in ALS. Here, our data suggests disrupted RNA trafficking in oligodendrocytes in both sporadic ALS cases and *C9orf72* ALS cases.

Using BaseScope^TM^, we undertook qualitative (spatial and subcellular localization) and quantitative examination of RNA trafficking in oligodendrocytes in ALS. ALS patient samples exhibited pathological accumulation of mRNAs both within the nucleus and in the oligodendrocyte processes/cytoplasm, with the patterns of accumulation mirroring the pattern of proteinopathy observed in both sALS and *C9orf72* cases (Supplementary Figures 1, 2). The *C9orf72* and sALS cases had increased nuclear localization of *MBP* mRNA and exhibited cytoplasmic aggregations of *MBP* mRNA implying both impaired nucleocytoplasmic transport as well as disrupted mRNA trafficking. Indeed, the concept of impaired trafficking of *MBP* mRNA in oligodendrocytes in ALS has been suggested recently, based on proportions of myelin protein expression (Lorente Pons et al., 2020) and our use of the highly sensitive BaseScope^TM^ technique allowed us to spatially verify this at an mRNA level. Further, it was suggested that the disruption to RNA trafficking correlated to protein pathology (Lorente Pons et al., 2020), an observation consistent with the pattern of *MBP* mRNA accumulation in both sALS and *C9orf72* ALS cases.

Impaired RNA trafficking was not limited to only *MBP.* We also identified a trend toward impaired *CAII* mRNA trafficking in sALS and *C9orf72* ALS patients compared to controls. Although, of itself, insufficient to make any firm conclusions it is of particular interest given that CAII plays an important role in the metabolic function of oligodendrocytes as a binding partner for MCT1 (32). MCT1 is a key transporter responsible for shuttling lactate out of oligodendrocytes to neurons and has previously been reported to be reduced in ALS patients (Lee et al., 2012; Philips et al., 2013). CAII is abundantly expressed in oligodendrocytes (Komoly et al., 1987), locally translated (Baron and Hoekstra, 2010) and is a binding partner of hnRNP A2 (Carson and Barbarese, 2005). Together these finding suggest a cell autonomous deficit of oligodendrocytes in ALS and would argue for further studies, including examination of metabolic dysfunction.

However, despite evidence of impaired mRNA trafficking, no difference in myelination between control, sALS or *C9orf72* cases was identified. Conflicting reports of myelination status in ALS have been reported. Whilst one report showed a significant decrease in MBP and CNPase protein levels in both the human motor cortex and lumbar spinal cord gray matter (Kang et al., 2013), another study conversely showed an increase in CNPase protein levels (Lee et al., 2012). A more recent study mirrored our findings by also showing no change to MBP protein levels (Lorente Pons et al., 2020) suggesting a compensatory mechanism within the oligodendrocytes allowing them to maintain myelination despite impaired RNA trafficking. In the well characterized SOD1^G93A^ mouse model of ALS, one study found decreased numbers of mature oligodendrocytes, increased oligodendrocyte precursors as well as lower MBP and CNPase protein levels yet also showed a lower g-ratio (which corresponds to thicker myelin sheaths) and no quantifiable difference in LFB staining of the lumbar spinal cord (Kang et al., 2013). Another study in this same mouse model showed neither a change to the number of mature oligodendrocytes, nor to MAG and CNPase protein levels, but showed a decrease in MBP protein at end stage disease (Philips et al., 2013). Crucially, these studies did not evaluate patient post-mortem tissue from SOD1 patients (spinal cord tissue may be especially informative), to assess the translational relevance of these *in vivo* findings, highlighting areas for future research.

Whilst we definitively show altered oligodendrocyte biology in ALS, there are some limitations to our study that deserve addressing. Access to, and ethical use of, human post-mortem tissue limited our sample size yet we were still able to achieve statistical power in our analyses. We predict that increasing our sample size would indeed strengthen findings with respect to altered RNA trafficking, given how stark the differences were between sALS and C9 ALS cases compared to control. Despite the small sample size, a strength of our study is that we have used deeply phenotyped cases that has allowed us to separate C9 ALS cases from sALS, which is important when assessing cellular biology given their distinct pathologies (particularly with respect to cellular localization of pathology) despite converging on similar disease phenotypes, like impaired RNA trafficking. We unfortunately were unable to co-label such pathologies with oligodendrocyte-specific markers. When assessing myelin biology using ALS patient post-mortem tissue, two studies have assessed the 18.5 kDa MBP isoform with one also assessing CNP levels (Kang et al., 2013) and the other also assessing PLP levels (Lorente Pons et al., 2020). We chose to only assess MBP as it is the only major myelin protein known to be locally translated; we have assessed two MBP isoforms, one that is locally translated and one that is not; unfortunately, it remains challenging to further clarify other MBP isoforms in human post-mortem tissue using standard Western blotting. Whilst previous studies focusing on oligodendrocytes have analyzed the lumbar spinal cord, we chose to focus on the motor cortex white matter where an oligodendrocyte deficit may be cell autonomous rather than secondary to Wallerian degeneration.

## Conclusion

Our findings, including the first ultrastructural examination of human myelination in ALS, suggest that there is no evidence of a myelination deficit in the motor cortex of ALS patients. Despite no evidence of abnormal myelination, oligodendrocytes have multiple other roles, many of which depend on local translation, thereby highlighting that a cell autonomous RNA trafficking deficit in oligodendrocytes may still have significant functional consequences. In fact, previous studies assessing Cnp1 loss of function in mice (33) demonstrated an uncoupling of myelination and axonal support roles, where molecules essential for axonal survival are not required for myelin assembly, implying that these functions are distinct and not interdependent. Consistent with this, we show that although trafficking of MBP is disrupted in in sALS and *C9orf72* ALS cases, there is no evidence of an ultrastructural deficit in myelination.

## Data Availability Statement

The raw data supporting the conclusions of this article will be made available by the authors, without undue reservation.

## Ethics Statement

The studies involving human participants were reviewed and approved by the East of Scotland Research Ethics Service Edinburgh Brain Bank Ethics Committee Academic and Clinical Central Office for Research and Development Medical Research Ethics Committee. Written informed consent was not provided because written informed consent for posthumous collection of tissue was provided by patients.

## Author Contributions

SKB, JMG, CS, and SC contributed to the study conception and design. SKB, JMG, BTS, KM, OGJ, ARM, and DS performed the material preparation, data collection, and analysis. CH and TS-J provided the tissue for EM imaging. AMI provided the antibodies for DPRs. All authors read and approved the final manuscript.

## Conflict of Interest

The authors declare that the research was conducted in the absence of any commercial or financial relationships that could be construed as a potential conflict of interest.

## Publisher’s Note

All claims expressed in this article are solely those of the authors and do not necessarily represent those of their affiliated organizations, or those of the publisher, the editors and the reviewers. Any product that may be evaluated in this article, or claim that may be made by its manufacturer, is not guaranteed or endorsed by the publisher.
