# Supplementary figures and images for: Dysregulation in Subcellular Localization of Myelin Basic Protein mRNA Does Not Result in Altered Myelination in Amyotrophic Lateral Sclerosis
Source: Front Neurosci. 2021 Sep 1;15:705306. doi: 10.3389/fnins.2021.705306 (PMC8440970; doi:10.3389/fnins.2021.705306)

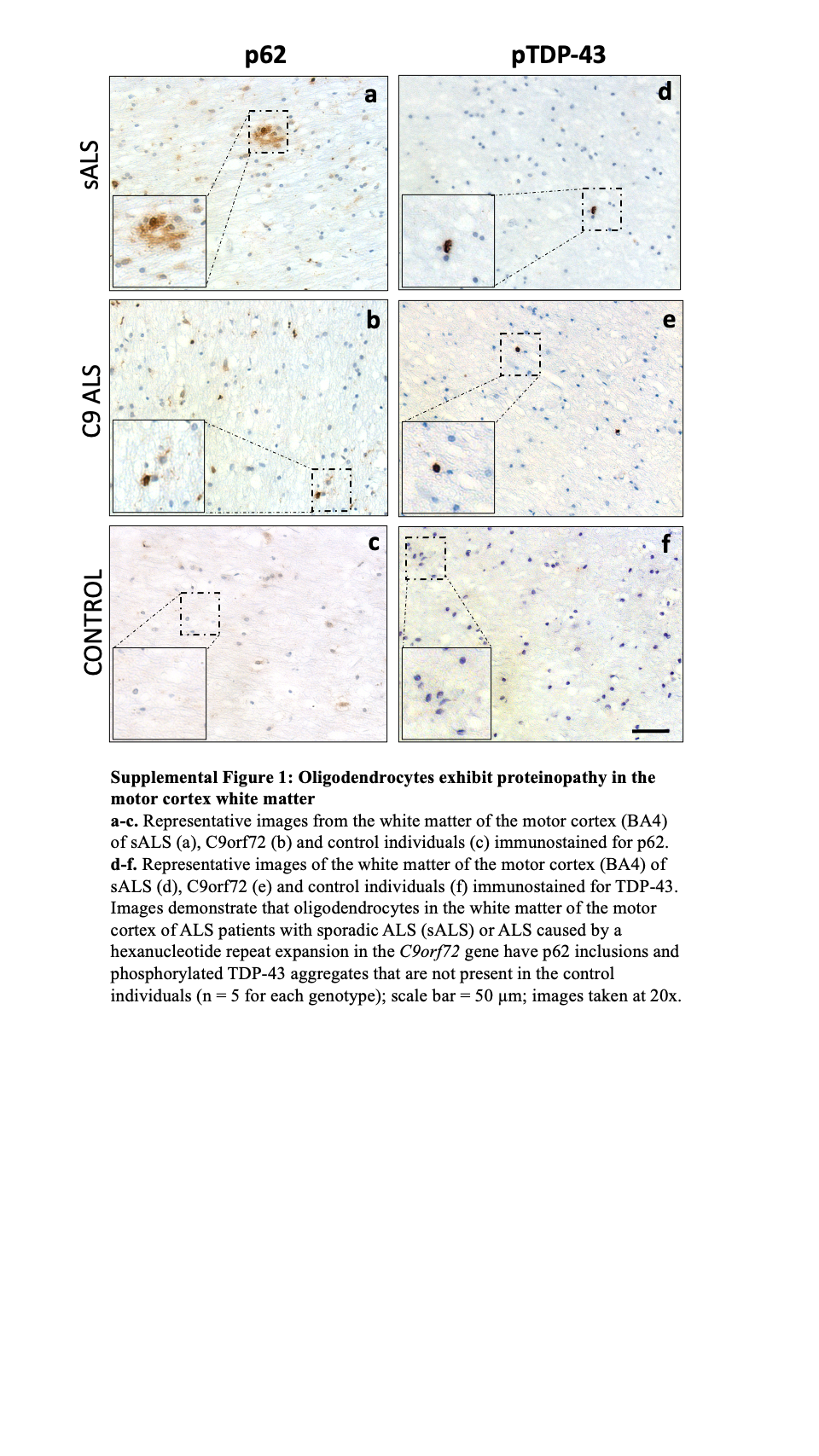

Supplement: Supplementary file 1 [file Image_1.TIFF]

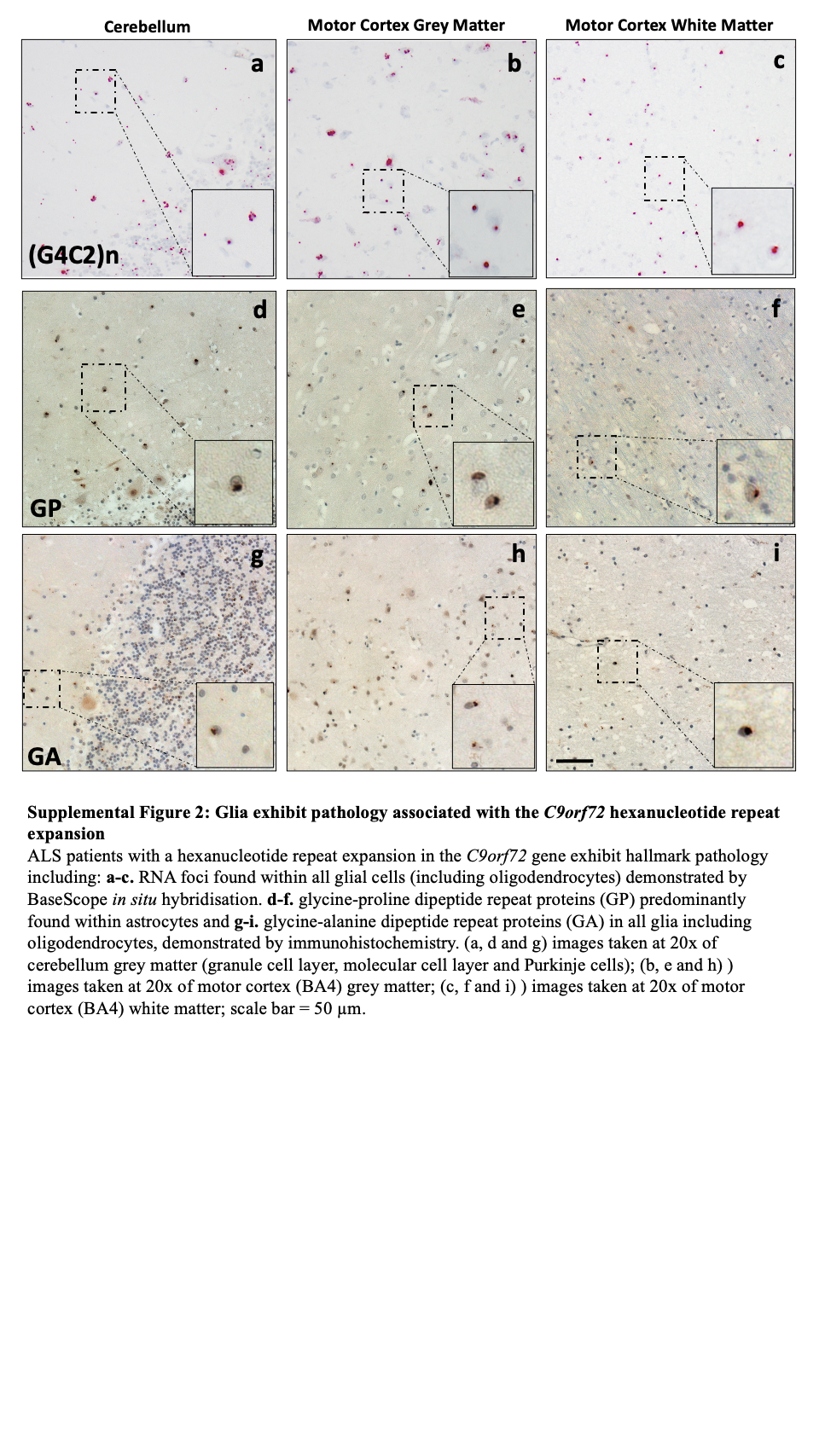

Supplement: Supplementary file 2 [file Image_2.TIFF]

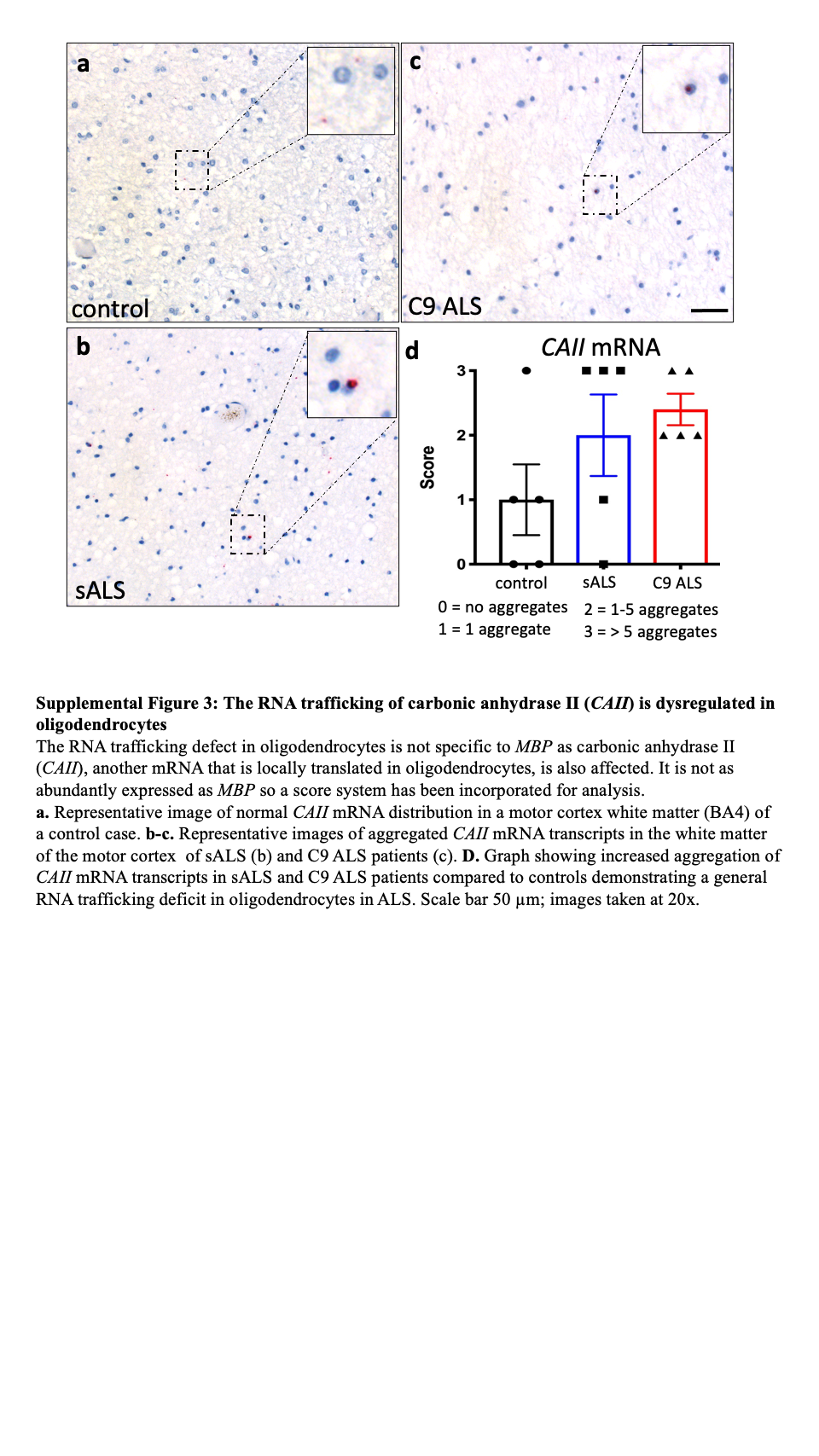

Supplement: Supplementary file 3 [file Image_3.TIFF]

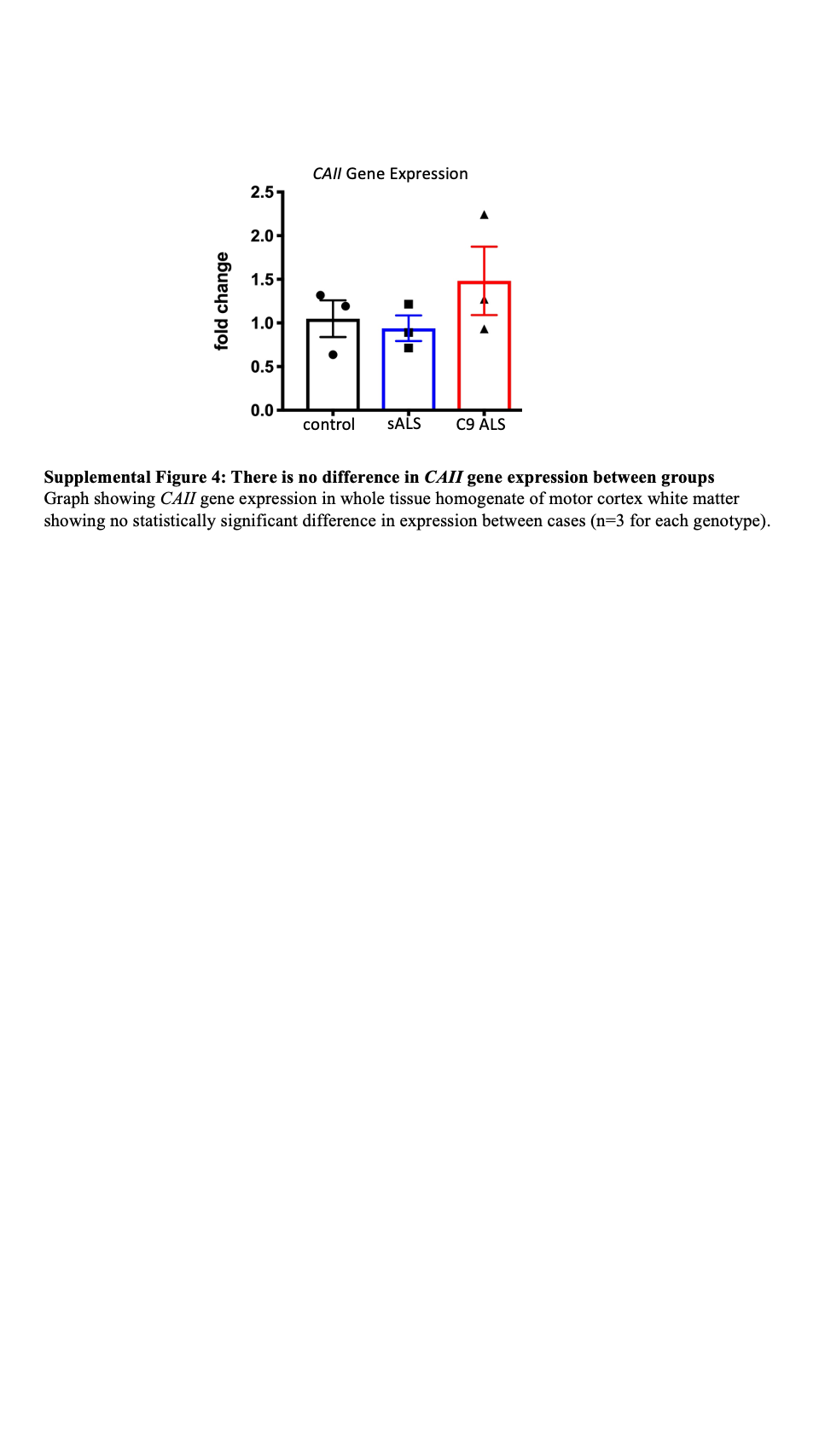

Supplement: Supplementary file 4 [file Image_4.TIFF]

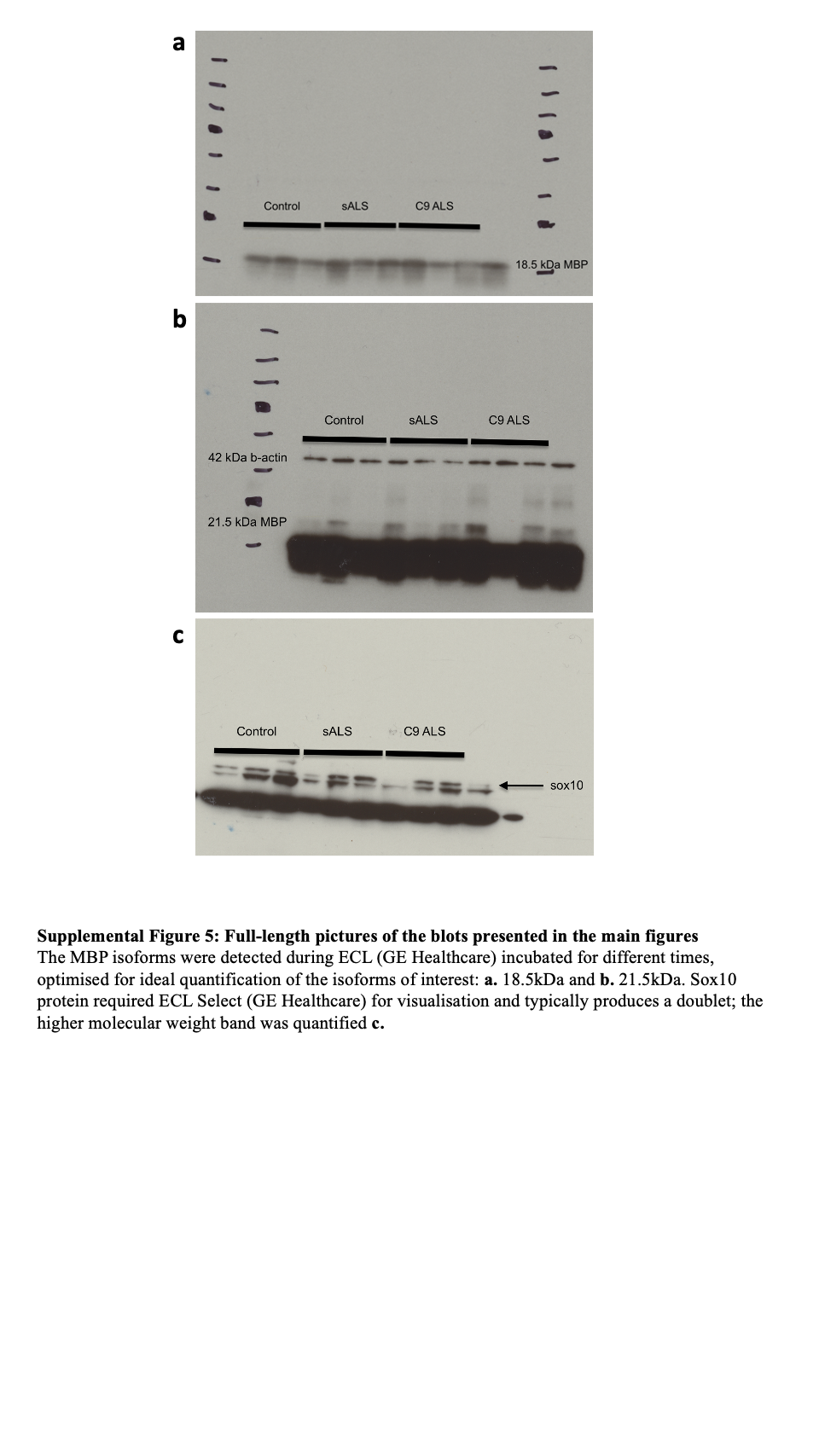

Supplement: Supplementary file 5 [file Image_5.tiff]

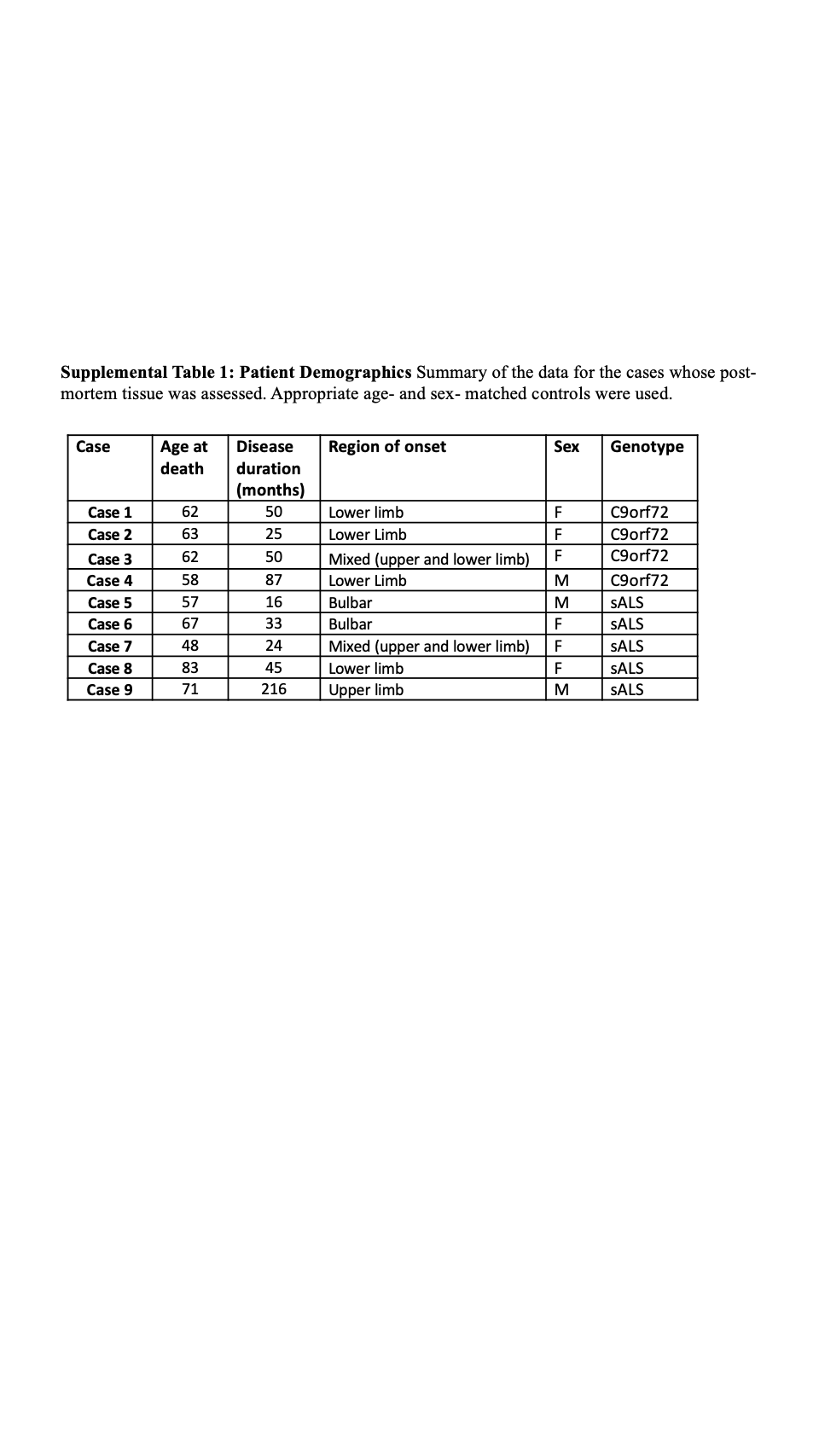

Supplement: Supplementary file 6 [file Image_6.TIFF]
